# Determinants of severe acute malnutrition among children under five years in a rural remote setting: A hospital based study from district Tharparkar-Sindh, Pakistan

**DOI:** 10.12669/pjms.342.14977

**Published:** 2018

**Authors:** Aurangzeb Sand, Ramesh Kumar, Babar T Shaikh, Ratana Somrongthong, Assad Hafeez, Dalpat Rai

**Affiliations:** 1 Aurangzeb Sand, Health Services Academy, Islamabad, Pakistan. Taluka Hospital, Nagarparkar, Sindh, Pakistan; 2 Ramesh Kumar, MBBS, MBA, MPH, PhD. Assistant Professor, Health Services Academy, Islamabad, Pakistan; 3 Babar T Shaikh, Health Services Academy, Islamabad, Pakistan; 4 Ratana Somrongthong, College of Public Health Sciences, Chulalongkorn University Thailand; 5 Assad Hafeez, Health Services Academy, Islamabad, Pakistan; 6 Dalpat Rai, Taluka Hospital, Nagarparkar, Sindh, Pakistan

**Keywords:** Pakistan, Poverty, Severe Acute Malnutrition, Sindh, Under Five Children

## Abstract

**Objectives:**

To understand and catalogue the specific determinants of this alarming rate of malnutrition among children of Tharparkar district, Sindh Pakistan.

**Methods:**

This was a hospital based analytical survey. Data was collected through a semi-structured questionnaire by interviewing mothers of the children (age 6-59 months), admitted in the hospital. Following WHO guidelines, weight and length/ height of 105 children were recorded. Study was conducted in District Headquarters Hospital, Tharparkar district of Sindh province.

**Results:**

Almost 48% children admitted in the hospital were identified with severe acute malnutrition. More males (55%) were malnourished as compared to females (45%). Maternal education, household income, family size, breastfeeding, vaccination status, and frequent infections were found to be significantly associated with the severe acute malnutrition.

**Conclusion:**

Specific interventions on promoting exclusive breastfeeding, vaccination, and timely health care seeking behaviors would definitely improve the outcomes. Nevertheless, sector wide approaches would be needed on girls’ education, poverty, and food security in the district in order to address the issue of malnutrition.

## INTRODUCTION

The word ’Malnutrition’ is generally used for both over nutrition and under nutrition cases; whereby the under nutrition means solely a deficiency of nutrition. More scientifically speaking, undernutrition includes being underweight for one’s age, too short for one’s age (stunted), dangerously thin (wasted), and deficient in vitamins and minerals (micronutrient malnutrition). 1 Food deprivation, lack of maternal education, inappropriate feeding and infections could be the major causes for the latter type of malnutrition.^2^ Malnutrition is a leading killer in children under five years.^3^ World Health Organization (WHO) defines Severe Acute Malnutrition (SAM) as a very low weight for height, by visible severe wasting, or by the presence of nutritional edema. Under nutrition in childhood, including fetal growth restriction, stunting, wasting, deficiencies of vitamin A and zinc, and suboptimal breastfeeding, have been causing 3.1 million child deaths annually, representing 45% of the total childhood mortality.^4^ Acute malnutrition is a serious public health problem, at times, and in regions reaching to the epidemic proportions. Worldwide, about 55 million under five children suffer from an acute malnutrition, and 26 million of them had been severely and acutely malnourished, particularly in sub-Saharan Africa and South Asia.^5^ Malnutrition is aggravated by diarrhea; one of the leading cause of under-five childhood mortality over last two decades. The risk of mortality is reported fifteen times higher, if pneumonia exists as a co-morbidity with severe malnutrition.^6^ Moreover, SAM is prevalent in areas affected by natural disasters and conflict, and thus requires immediate action to save the precious lives of children.^7^

Malnutrition is a major public health issue in Pakistan, and has escalated with a growing population.^8^ Country failed to meet its child mortality reduction targets set in MDGs, partly due to security issues, and natural disasters such as earthquake, flood, and droughts. National Nutrition Survey (NNS) 2011 noted that these inevitable factors rendered a large population homeless, hunger-struck and exposed to infections and malnutrition.^9^ A research conducted in Punjab province shows that illiteracy, large family size, lack of breastfeeding, and poverty are the main factors responsible for malnutrition in under-5 children.^10^ Another study from Sindh province revealed same reasons besides an additional factor i.e. recurrent diarrhea.^11^ In northern areas of Pakistan, restricted mobility of women, poverty and limited knowledge of child’s illness have been documented as major determinants of child health outcomes.^12^ According to Pakistan Demographic and Health Survey (PDHS) 2012-13, in rural parts of Sindh, 63% of children were stunted (amongst which 40% were severely stunted), 48% were underweight and 14% are wasted.^13^ With this state of affairs, it becomes critical to do a fresh stock taking of the current situation, and subsequently suggest practical and impact interventions to the program managers and the decision makers in the provincial health department.

The main objective of this study was to understand and catalogue all the determinants and their association with the SAM among children in the district Tharparkar, which stands out in terms of SAM statistics. Having developed this understanding, it is envisaged that more focused and selected interventions will help in reducing this alarming rate of SAM in the area. Therefore, these findings will be shared with the program managers, research community and the policy makers for deciding on the future roadmap to confront SAM in the province, and especially in the ditrict Tharparkar.

## METHODS

### Study Setting

This study was conducted in the district Tharparkar in Sindh province. Around 71% population habitat is rural, devoid of the most basic amenities of life.^14^ Life is harsh for people living in this rough terrain. Scorching heat in summer, shortage of food, unsafe and saline drinking water and hard to access primary health care facilities have had a hostile effect on the daily life and well-being of the communities. People are generally poor (living under US$2 a day), with limited sources of livelihood, mainly agriculture and livestock. Food insecurity is frequently seen because of severe drought like conditions especially during summer season. in As a result, under nutrition is a common problem among children as well as women.^15^

### Study Population

Our study universe comprised all children of age 6-59 months admitted during the month of October 2016 in the District Headquarters Hospital, Tharparkar. This is a secondary level of care facility which provides pediatric services including out/in patient department, and a neonatal nursery. It is also a referral facility for all the primary healthcare facilities in the entire district. Malnourished children who were suffering from other associated illnesses such as congenital heart diseases, metabolic disorder, renal failure and endocrine disorder were excluded from the study.

### Study Design

This cross sectional analytical survey was carried out at the pediatric ward of the district headquarters hospital in Mithi town, district Tharparkar, Sindh province. The sample size was calculated using the formula: (Z^2^×p×q)/d^2^, where ‘Z’ represents 95% confidence level, and is valued at 1.96, ‘p’ = 0.066 based on a previously reported prevalence of 6.6% of severe acute malnutrition in National Nutrition Survey 2011 and Pakistan Demographic Health Survey 2012-13, ‘q’ = 1-p, and ‘d’ representing 5% confidence interval (valued at 0.05) was 95. With a 10% additional sample to cover likely refusals, we studied 105 children admitted in the hospital. Data was collected from 1^st^ to 31^st^ October 2016.

### Research Instrument

A semi structured questionnaire was adapted from the WHO & UNICEF instrument for asking questions from mothers of the admitted children.^16^ Tool was pre tested and piloted at another hospital in the adjacent district Nagarparkar. After necessary modifications, the semi structured questionnaire was finalized. It included variables on socio-demographic characteristics of the child and family e.g. age, sex of child, birth order, maternal education (schooling at least till grade 5), and household income. The minimum wages were taken as Rs12000 per month.^17^ Distance of nearest health facility was also recorded as a variable of interest. Furthermore, practice of exclusive breastfeeding, vaccination status (self reported), start of weaning, any infections like diarrhea and pneumonia were documented. The weight and length/ height were measured in accordance of WHO WHZ score charts. Weight was recorded on RGZ – 20 automatic weighing scale with 100 gram gradients, height of children of more than two years of age was recorded through stadiometer, and length of children of less than two years of age was recorded through infantometer. The WHZ Score between ±2 SD was defined as a normal nutritional status, WHZ Score between -2 and -3 SD was defined as moderate acute malnutrition and WHZ Scores <-3 SD was classified as severe acute malnutrition.

### Data Analysis

Data was analyzed with SPSS version 20.0. The association of SAM (present or absent) with various independent variables was computed with chi square and p values were computed. The statistically significant results were highlighted with a p-value <0.05.

### Ethical Considerations

The study protocol was approved by the Institutional Review Board of Health Services Academy, Islamabad. A written permission to conduct the study was obtained from the District Health Officer Tharparkar and by Civil Surgeon DHQ Hospital Mithi. Written informed consent was obtained from the mothers and caregivers of the children who were interviewed for data collection.

## RESULTS

Total 105 children (age between 6-59 months) were included, and the sample comprised 55.2% male and 44.8% female children. With regard to age, 35.2% were 6-12 months old, 44.8% were 13-36 months, and 20% were 37-59 months of age. The assessment of nutritional status of the children admitted in the hospital revealed that 21% children were normal, 31.4% were suffering from moderate acute malnutrition, and 47.6% children manifested severe acute malnutrition. Table-I and Table-II show the weight and height/ length of children in age category.

**Table-I:** Weight of children in age category.

Child’s Age	Number	Minimum Weight	Maximum Weight	Mean Weight	Std. Deviation
6-12 months	37	3.00	9.00	5.7189	1.36215
12-36 months	47	3.40	12.00	7.6553	1.51023
36-59 months	21	5.20	15.00	9.8190	2.16463

**Table-II:** Height / length of children in age category.

Height	Number	Minimum HT	Maximum HT	Mean HT	Std. Deviation
6-12 months	37	56.00	78.00	66.5541	5.16906
12-36 months	47	55.00	92.00	76.9255	7.29501
36-59 months	21	53.00	110.00	89.5714	12.12671

### Association of socio-demographic characteristics with SAM

We found that the age and sex of the child was not associated with SAM status. However, maternal education status was significantly associated, and SAM was found higher in the children of illiterate mothers. Moreover, family size and household income were also found to be significantly associated with SAM, as shown in Table-III.

**Table-III:** Association of socio-demographic characteristics with SAM.

Variable	Frequency	Percent	Sam Present	Sam Absent	p value
**Age of child**					

6m-12m	37	35.2	19	18	0.606
12m-36m	47	44.8	23	24	
36-59m	21	20	8	13	

**Sex of child**					

Male	58	55.2	31	27	0.184
Female	47	44.8	19	28

**Birth order**					

≤3	64	61	30	34	0.849
>3	41	39	20	21

**Family size**					

<5	42	40	9	33	0.001 *
≥5	63	60	41	22

**Maternal educational status**					

Educated	19	18.1	01	18	0.04*
Uneducated	86	81.9	49	37

**Household income**					

≥Rs. 12000	22	21	01	21	<0.001*

**Table-IV:** Table shows an association of other study variables with SAM.

Variable	Frequency	Percent	Sam Present	Sam Absent	p value
**Exclusive breastfeeding**					
Yes	44	41.9	4	40	0.001*
No	61	58.1	46	15	(0.010-0.106)

**Weaning**					

Timely	32	30.5	1	31	0.001*
Untimely	73	69.5	49	24	(0.002-0.123)

**Vaccination status**					

Complete	55	52.4	8	47	0.001*
Incomplete	50	47.6	42	8	(0.011-0.094)

**Diarrhea**					

Not frequent	50	47.6	7	43	0.001*
Frequent	55	52.4	43	12	(0.016-0.126)

**Pneumonia**					

Not frequent	54	51.4	7	47	0.0001*
Frequent	51	48.6	43	8	(0.009-0.083)

**Distance to nearest health facility**					

<20km	22	21	01	21	<0.690 (0.536-2.566)

## DISCUSSION

This hospital based study revealed a relatively higher rates of SAM (47.6%) in this part of province; as compared to the malnutrition reported for Sindh province by NNS 2011 showing 21-23%, and PDHS 2012-13 showing 14%, although latter two surveys were based on community samples.^9^,^13^ Among all districts of Sindh, caloric poverty level was seen as highest in rural Tharparkar, an arid region of province, where close to two-thirds population was food anxious.^18^ Our results concur with studies conducted in Bangladesh and India, where mothers’ education status and poor breastfeeding practices were found to be associated with SAM.^19^,^20^ Parental education especially of the mother surely determines the nutrition status of young children in developing countries; and our study has once again validated this fact.

Studies from Nigeria and India show association of child vaccination with SAM.^2^,^20^ Same association has been documented for Pakistan^21^, and is reiterated by our study results too. Other significantly associated factors such as frequent episodes of diarrhea and pneumonia in children with SAM have been reported in other studies in Pakistan as well as in the neighboring region.^10^,^22^,^23^ Exclusive breastfeeding is one of the most useful and inexpensive intervention to reduce child mortality and make the child healthy and prevent from diseases such as diarrhea and malnutrition.^24^ SAM was found to be significantly associated with breastfeeding being non-exclusive in our findings, and other authors also reported the same fact.^10^,^11^ Recent data shows that exclusive breastfeeding rate is declining in Pakistan.^25^,^26^ Similarly, timing of initiation of weaning is an important determinant of SAM.^13^,^27^. Our finding of the household income and level of poverty associated with SAM among child is also in line with other studies which revealed more obvious malnutrition in families where monthly household income was low.^12^,^20^,^28^ Nutrition education of expecting mothers must be ensured as a routine activity of the ante-natal visits in the health facility.^29^ Moreover, the lady health workers who are the strength of primary health care in Pakistan, and who cover the population door to door, could be instrumental in identifying and treating the cases of SAM in the community.^30^

## CONCLUSION

The study concludes that this grave issue of SAM among children under five needs to be addressed with multi-faceted approach and multi-pronged strategies. Since mothers’ education and household poverty were found to be significantly associated, these factors need to be worked upon by investing in girls’ education, and provision of safety nets to serve the poorest of the poor. Moreover, immunization program, family planning program and breast feeding promotion need to integrate their respective strategies to address the issue of malnutrition in the district of Tharparkar.

### Author’s Contribution

**AS, RK:** Designed and performed the study.

**AZ, RS:** Did statistical analysis and editing of manuscript.

**BTS, DR:** Did data collection and writing of manuscript.
